# Lessons Learned From the Historical Trends on Thrombolysis Use for Acute Ischemic Stroke Among Medicare Beneficiaries in the United States

**DOI:** 10.3389/fneur.2022.827965

**Published:** 2022-03-04

**Authors:** Tong Meng, Amber W. Trickey, Alex H. S. Harris, Loretta Matheson, Sarah Rosenthal, Abd Al-Rahman Traboulsi, Jeffrey L. Saver, Todd Wagner, Prasanthi Govindarajan

**Affiliations:** ^1^Department of Emergency Medicine, Stanford University, Stanford, CA, United States; ^2^Stanford–Surgery Policy Improvement Research & Education Center (S-SPIRE), Department of Surgery, Stanford, CA, United States; ^3^Veterans Affairs Health Services Research and Development Center for Innovation to Implementation, Palo Alto Veterans Affairs Health Care System, Menlo Park, CA, United States; ^4^Stanford School of Medicine, Stanford, CA, United States; ^5^Comprehensive Stroke Center and Department of Neurology, David Geffen School of Medicine, University of California, Los Angeles, Los Angeles, CA, United States

**Keywords:** acute ischemic stroke, intravenous thrombolysis, temporal trends, disparities of care, stroke registry

## Abstract

**Background:**

The most recent time trends on intravenous thrombolysis (IVT) utilization for acute ischemic stroke was reported in 2011 using the Get with the Guidelines. Our objectives are to assess and validate the change in IVT utilization through 2014 in a national sample of Medicare beneficiaries and to examine the effect of patient, stroke center designation, and geography on IVT utilization.

**Methods:**

We built a comprehensive national stroke registry by combining patient-level, stroke center status, and geographical characteristics, using multiple data sources. Using multiple national administrative databases from 2007 to 2014, we generated a mixed-effect logistic regression model to characterize the independent associations of patient, hospital, and geographical characteristics with IVT in 2014.

**Results:**

Use of IVT increased consistently from 2.8% in 2007 to 7.7% in 2014, *P* < 0.001. Between group differences persisted, with lower odds of use in patients who were ≥86 years (aOR 0.74, 95% CI 0.65–0.83), Black (aOR 0.73, 95% CI 0.61–0.87), or treated at a rural hospital (aOR 0.88, 95% CI 0.77–1.00). Higher odds of use were observed in patients who arrived by ambulance (aOR 2.67, 95% CI 2.38–3.00), were treated at a hospital certified as a stroke center (aOR 1.96, 95% CI 1.68–2.29), or were treated at hospitals located in the most socioeconomically advantaged areas (aOR 1.27, 95% CI 1.05–1.54).

**Conclusions:**

Between 2007 and 2014, the frequency of IVT for patients with acute ischemic stroke increased substantially, though differences persisted in the form of less frequent treatment associated with certain characteristics. These findings can inform ongoing efforts to optimize the delivery of IVT to all AIS patients nationwide.

## Introduction

Stroke is the leading cause of serious long-term disability and the fifth leading cause of mortality in the US (1). Reperfusion to minimize ischemic stroke-related disability and mortality is achieved through intravenous thrombolysis (IVT) treatment and through mechanical thrombectomy procedures for large vessel strokes (2). Despite the clear benefit of IVT use in ischemic stroke, at the start of the twenty-first century only 1–2% of ischemic stroke patients in the United States received IVT (3, 4). Over the next decade, there was a steady but slow increase in IVT use as reported by previous studies using registry data and Medicare claims data (5, 6). The most recent national assessment of temporal trends from 2003 to 2011 was reported by Get with the Guidelines study team. In the early 2010s, the frequency of IVT use remained suboptimal, with more than one-fifth of patients documented as fully eligible for treatment not receiving thrombolytic therapy even at the most committed, registry-participating hospitals (6). Notably, older patients, Non-white patients, and patients who arrived at emergency departments (ED) using private transport were particularly less likely to receive IVT (6). In addition, a majority of acute ischemic stroke patients during these time periods were ineligible for IVT because they arrived at hospital beyond the abbreviated time window in which therapy is beneficial, with late arrival associated with unawareness of stroke warning signs and lack of close geographic access to a treating stroke center (7–9).

During this early 2010s time period, patient characteristics of race-ethnicity, sex, and socioeconomic status were also found to exacerbate reduced IVT rates. Black, Hispanic, and lower median income patients were less likely to be treated at a high volume thrombolytic hospital and to receive thrombolytic therapy (10–12). Older patients and women were also less likely to receive thrombolytic therapy (12). At the hospital level, longer waiting times in the ED, language barriers, fewer race-concordant physicians, and other unrecognized biases within the healthcare system have been suggested to contribute to the differences in emergency stroke treatment (13, 14).

The evolution of the delivery of thrombolytic therapy for acute ischemic stroke in the United States subsequent to the 1999–2010 period has not been well-delineated. Moreover, few studies in any time period examined a large, fully generalizable, nationally representative dataset. In this study, we examined thrombolysis patterns in the early to mid-part of the last decade. While our data pre-dates the thrombectomy era and could have changed over the years, we believe that most recent efforts have been directed toward increasing thrombectomy and therefore, thrombolysis patterns may not have seen any major shifts over the years. Also, due to lag in availability of national data, examination of trends for recent years may not be feasible. Accordingly, the objectives of this study were to examine IVT utilization among Medicare beneficiaries over an 8-year period from 2007 to 2014 and to examine the associations of patient and hospital-level characteristics with IVT utilization.

## Methods

The study was approved by the Centers for Medicare and Medicaid Services, the Research Data Assistance Center (ResDAC), and the Stanford University Institutional Review Board.

### Data Sources

In this observational study, we analyzed a 20% national sample of Medicare administrative claims data from outpatient files, submitted by institutional providers, and carrier files, submitted by professional providers, ambulance providers and free-standing facilities. We also used the Medicare Provider Analysis and Review (MedPAR) file, which aggregates inpatient and skilled nursing facility claims information at the visit level (15), and the Medicare Master Beneficiary Summary File (MBSF), an enrollment database that contains the patient demographic and enrollment information (16). We evaluated data for the 8-year period from 2007 to 2014. To capture hospital characteristics, we included data from the Provider of Services Current File (POS), which contains information for CMS certified hospitals/facilities (17). Stroke center certification data were derived from the Joint Commission (18), Healthcare Facilities Accreditation Program (HFAP) (19) and Det Norske Veritas (DNV) (20).

### Study Cohort

We created a cohort of patients aged ≥ 66 years who received care in the ED and were subsequently admitted with a primary diagnosis of ischemic stroke (International Classification of Diseases, Ninth Revision, Clinical Modification, ICD-9-CM: 433.x1, 434.x1, 436). Age cutoff was chosen to ensure at least one full year of baseline health information prior to the stroke event. Within this cohort, we studied the first acute ischemic stroke hospitalization for each patient; ED visits and hospital admissions were constructed using the MedPAR file and outpatient claims. Inpatient records were determined to be associated with a prior ED visit if: 1) the MedPAR ED charge amount (variable: ER_AMT) was >$0, or 2) emergency services were reported in outpatient claims ≤ 2 days prior to MedPAR inpatient admission date. Further, to be included in the study cohort, we required patients to have been continuously enrolled in Medicare Part A and Part B (not Part C) for at least 1 year prior to the hospitalization to facilitate ascertainment of comorbidities. Patients with missing demographic information (age, sex, race/ethnicity) or missing information to calculate the hospital Area Deprivation Index (ADI) were excluded from the analysis. With this criteria, 2.3% of the overall sample were excluded

### Covariates

We obtained patient-level characteristics such as age, sex, race, and ethnicity from the Medicare Beneficiary Summary File. Specifically, patients were categorized into three age groups (66–75 years, 76–85 years, 86 years and above) and two sex groups (male and female); race-ethnicity categories were directly extracted from the CMS administrative database, including White, Black, Asian, Hispanic, North American Native and other. The race-ethnicity information in CMS records was obtained from the Social Security Administration records and further refined by algorithms generated by Research Triangle (21) Institute (RTI).

Patient residence mailing zip code from the Medicare Beneficiary Summary File was used to construct urban vs. rural classification. Specifically, using the 2010 Census classification, we defined zip codes with >50% of the population living in a rural area as “rural”; otherwise, the zip code was considered urban (22). Zip code was also used to designate patient residence census region (Midwest, Northeast, South, West, US Territories).

Other patient-level data include patient comorbidity and mode of transportation. Patient comorbidity was classified by the modified Charlson Comorbidity Index (Charlson score) using information collected from all care settings (inpatient, outpatient, carrier) (23, 24). Mode of transportation used by the patient to arrive at the ED [emergency medical services (EMS) vs. other] was identified using the Healthcare Common Procedure Coding System (HCPCS) codes using outpatient and carrier claims data (HCPCS codes A0426-A0434) (25).

Hospital-level characteristics include stroke certification status, hospital size, hospital Area Deprivation Index and the proportions of patients from racial or ethnic minority groups treated at the hospital. Stroke certification status indicates whether the hospital was capable of providing IVT treatment (thrombolysis-capable center) at time of the stroke hospitalization.

For hospital size, we obtained the number of beds from the POS file to create categories based on the number of beds (0–100 beds, 101–200 beds, 201–300 beds, 401–500 beds, ≥ 500 beds). Zip codes of hospitals were obtained from the POS file to assign hospital Area Deprivation Index, which is a validated measure of neighborhood socioeconomic status taking into account income, education, employment and housing quality provided at zip code level (26). Area Deprivation Index socioeconomic areas were classified in four categories by quartile, from the most advantaged (1st to 25th percentile) to the most disadvantaged (76th to 100th percentile).

Finally, we constructed proportion of stroke patients by race-ethnicity (White, Black, Asian, Hispanic, North American Native, and other) treated at the hospitals to reflect composition of the patient population hospitals serve.

### Outcome

The primary outcome was IVT, identified using ICD-9-CM procedure code 99.10 or Current Procedural Terminology (CPT) code 37195.

### Statistical Analysis

We used the Cochran-Armitage trend test to assess for temporal trends in IVT utilization over the 8-year study period. Bivariate associations between IVT and covariates of interest were assessed using chi-square tests. To estimate the contributions of patient and hospital characteristics to IVT utilization in the most recent year of observation, we calculated a mixed-effects hierarchical logistic regression model with random intercepts for hospital-level clustering and limited the analysis to the last year of data (2014). Two-tailed *P*-values < 0.05 were considered statistically significant. All analyses were performed using SAS v.9.4 (Cary, NC).

## Results

### Sample Characteristics

The 20% national sample yielded 228,007 acute ischemic stroke patients for the time trend analysis (entire study cohort, 2007 to 2014) and 28,434 acute ischemic stroke patients for the multivariable mixed-effects logistic regression analysis (final study year cohort, 2014).

Demographic and clinical characteristics of the 2007–2014 entire study cohort (*n* = 228,007) analyzed for time trend analysis are shown in Table 1. The 2014 final study year cohort (*n* = 28,434) analyzed for patient and hospital characteristics independently associated with IVT treatment is shown in Table 2. The characteristics of the two cohorts and subgroup relationships to IVT were similar. The 2014 final study year cohort differed from the 2007–2014 entire study cohort with a lesser percentage of patients who were women (57.8 vs. 60.0%) and patients aged between 76 and 85 (38.2 vs. 50.6%). The 2014 final study year cohort was predominantly White, predominantly urban, and predominantly characterized by a high comorbidity burden (≥2 comorbidities). Nearly two-thirds of patients were transported by EMS for the stroke episode.

**Table 1:** IVT from 2007 to 2014 for medicare patients by patient subgroup.

	**2007**	**2008**	**2009**	**2010**	**2011**	**2012**	**2013**	**2014**
**Age**								
66 to 75	267 (3.10)	340 (4.04)	324 (4.11)	422 (5.13)	464 (5.64)	535 (6.43)	637 (7.37)	722 (7.98)
76 to 85	390 (3.04)	467 (3.75)	503 (4.27)	635 (5.49)	611 (5.38)	679 (6.19)	777 (7.20)	857 (7.88)
86 and above	183 (2.11)	230 (2.76)	297 (3.56)	361 (4.28)	352 (4.08)	457 (5.27)	555 (6.56)	592 (6.95)
**Sex**								
Male	339 (2.87)	425 (3.70)	439 (4.04)	592 (5.22)	599 (5.29)	692 (6.16)	836 (7.39)	962 (8.01)
Female	501 (2.73)	612 (3.46)	685 (4.00)	826 (4.89)	828 (4.90)	979 (5.85)	1,133 (6.83)	1,209 (7.36)
**Race-ethnicity**								
White, Non-Hispanic	736 (2.85)	933 (3.73)	996 (4.15)	1,240 (5.13)	1,271 (5.27)	1,441 (6.05)	1,731 (7.26)	1,894 (7.80)
Black, Non-Hispanic	67 (2.18)	76 (2.57)	83 (2.92)	112 (3.96)	103 (3.63)	136 (4.81)	145 (5.24)	168 (5.92)
Hispanic	17 (3.31)	16 (3.17)	20 (4.39)	25 (4.00)	19 (4.00)	29 (6.43)	32 (7.46)	42 (10.05)
Asian	14 (3.47)	<11 (<3.03)^a^	20 (5.39)	26 (6.58)	20 (4.58)	30 (6.77)	41 (8.97)	41 (8.70)
American Native	0 (0.00)	0 (0.00)	0 (0.00)	<11 (<9.91)	<11 (<11.11)	<11 (<10.09)	<11 (<10.78)	<11 (<9.73)
Other	<11 (<4.44)	<11 (<4.70)	<11 (<4.42)	11 (4.10)	11 (4.38)	27 (8.85)	15 (5.14)	25 (7.96)
**Charlson score**								
Charlson score ≤ 1	64 (2.24)	68 (2.68)	64 (2.64)	93 (3.76)	72 (3.14)	97 (4.34)	107 (4.72)	121 (5.13)
Charlson score ≥ 2	776 (2.84)	969 (3.64)	1,060 (4.14)	1,325 (5.14)	1,355 (5.23)	1,574 (6.12)	1,862 (7.27)	2,050 (7.86)
**Geographic location**								
Urban	713 (2.94)	903 (3.84)	999 (4.43)	1,228 (5.38)	1,251 (5.48)	1,436 (6.40)	1,682 (7.51)	1,831 (8.04)
Rural	127 (2.15)	134 (2.36)	125 (2.30)	190 (3.51)	176 (3.28)	235 (4.26)	287 (5.22)	340 (5.99)
**Census region**								
Midwest	203 (2.52)	274 (3.61)	291 (4.00)	325 (4.57)	354 (4.99)	425 (6.02)	453 (6.59)	500 (7.19)
Northeast	214 (3.68)	274 (5.02)	247 (4.75)	286 (5.43)	287 (5.57)	336 (6.69)	394 (7.69)	472 (8.70)
South	286 (2.41)	344 (2.93)	394 (3.51)	562 (4.95)	515 (4.50)	603 (5.27)	722 (6.30)	793 (6.91)
West	137 (3.18)	145 (3.37)	192 (4.55)	245 (5.49)	270 (6.04)	306 (6.97)	399 (9.07)	405 (8.92)
**Mode of arrival**								
EMS transport	670 (3.64)	817 (4.60)	931 (5.36)	1,165 (6.63)	1,161 (6.57)	1,382 (7.93)	1,624 (9.23)	1,774 (9.86)
Other means of transportation	170 (1.45)	220 (1.93)	193 (1.81)	253 (2.37)	266 (2.52)	289 (2.74)	345 (3.35)	397 (3.80)

a *Cells expressing imprecise measurements are due to CMS small cell suppression policy. (https://www.hhs.gov/guidance/document/cms-cell-suppression-policy). Center for Medicare and Medicaid Services has set minimum cell sizes to protect the confidentiality of Medicare and Medicaid beneficiaries by avoiding the release of information that can be used to identify individual beneficiaries and therefore, we report cell sizes that comply with the requirements. When a small cell is present, two cells in the category must be masked to eliminate the mathematical derivation of the small cell*.

**Table 2:** Characteristics of patients admitted in 2014 by treatment status.

	**Patients, No. %**				
**Characteristics**	**No IVT** ***N*** **=** **26,263**		**IVT** ***N*** **=** **2,171**		***P*-value**
	***N***	**%**	***N***	**%**	
**Sex**					
Male	11,044	42.05%	962	44.31%	<0.001
Female	15,219	57.95%	1,209	55.69%	
**Age group**					
66 to 75	8,320	31.68%	722	33.26%	<0.001
76 to 85	10,017	38.14%	857	39.47%	
86 and above	7,926	30.18%	592	27.27%	
**Race**					
White	22,387	85.24%	1,894	87.24%	<0.001
Black	2,669	10.16%	168	7.74%	
Asian	430	1.64%	41	1.89%	
Hispanic	376	1.43%	42	1.93%	
American native	112	0.43%	<11^a^	<0.51%	
Other	289	1.10%	>15	>0.70%	
**Charlson comorbidity**					
Charlson score ≤ 1	2,236	8.51%	121	5.57%	<0.001
Charlson score ≥ 2	24,027	91.49%	2,020	93.04%	
**Geography**					
Urban	20,929	79.69%	1,831	84.34%	<0.001
Rural	5,334	20.31%	340	15.66%	
**Census region**					
Midwest	6,455	24.58%	500	23.03%	<0.001
Northeast	4,951	18.85%	472	21.74%	
West	4,135	15.74%	405	18.65%	
South	10,681	40.67%	>783	>36.07%	
US territories	41	0.16%	<11	<0.51%	
**EMS utilization**					
Ambulance users	16,222	61.77%	1,774	81.71%	<0.001
Other means of transportation	10,041	38.23%	397	18.29%	

a *Cells expressing imprecise measurements are due to CMS small cell suppression policy. (https://www.hhs.gov/guidance/document/cms-cell-suppression-policy). Center for Medicare and Medicaid Services has set minimum cell sizes to protect the confidentiality of Medicare and Medicaid beneficiaries by avoiding the release of information that can be used to identify individual beneficiaries and therefore, we report cell sizes that comply with the requirements. When a small cell is present, two cells in the category must be masked to eliminate the mathematical derivation of the small cell*.

### IVT Use Over Time

**Figure 1:**
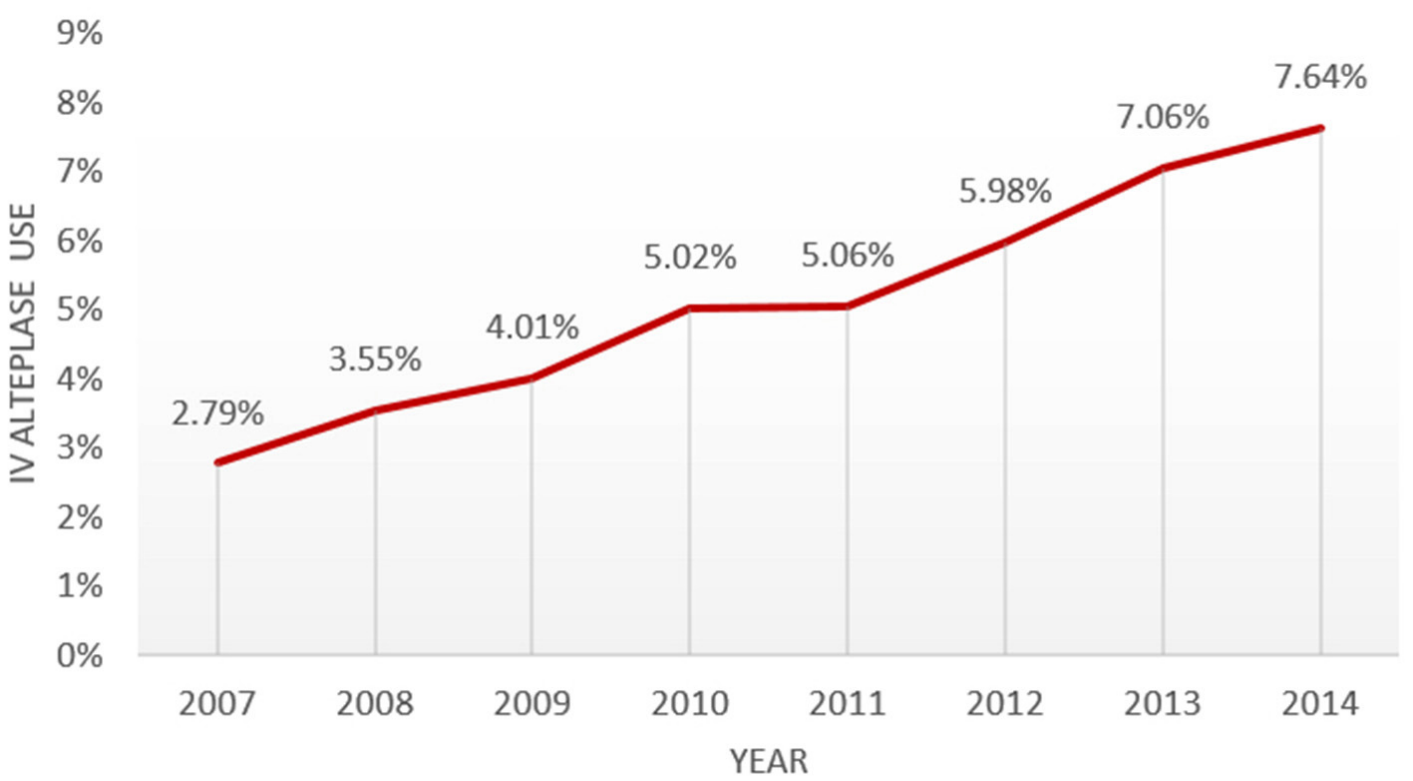
Temporal trend from 2007 to 2014 in IVT use in a national sample of medicare patients hospitalized for acute ischemic stroke.

Over the 8-year study period, among entire study cohort (2007–2014) patients 66 years and older hospitalized for acute ischemic stroke, the proportion treated with IVT increased from 2.8% in 2007 to 7.7% in 2014 *(P*<*0.0*01, Figure 1). This positive trend was observed in most subgroups of patients (Table 1).

### Associations of Patient and Hospital Characteristics With IVT Use

In bivariate analysis of both the entire study cohort (2007–2014) and the final study year cohort (2014), patient characteristics associated with higher frequency of IVT use were younger age, male sex, White, Asian, and Hispanic race-ethnicity, and the presence of more comorbidities. Higher frequency of IVT use were also associated with residing in urban areas and living in the West and Northeast of the United States. Patients transported by EMS were more likely to receive IVT when compared to those who arrived by other means of transport (Table 2 and Supplementary Table 1).

In bivariate analyses of hospital characteristics, IVT use was greater in hospitals certified as stroke centers (thrombolysis-capable), in hospitals with >100 beds, and in hospitals located in more advantaged areas defined by the Area Deprivation Index (Table 3).

**Table 3:** IVT rates in hospitals with different characteristics (2014).

**Characteristics**	**Overall** ***N* = 3,132**	**IVT rate**	**Standard** **deviation**	***P-*** **value**
**Stroke certification status**				<0.001
Never certified	1,738	1.5%	6.5%	
Ever certified	1,394	8.7%	11.8%	
**Hospital size**				<0.001
0 to 100 beds	2,139	0.4%	2.1%	
101 to 200 beds	871	2.3%	4.1%	
201 to 300 beds	566	3.6%	4.1%	
301 to 400 beds	354	5.5%	5.3%	
401 to 500 beds	193	5.6%	4.4%	
More than 500 beds	355	7.2%	4.9%	
**Hospital ADI**				<0.001
Most advantaged	440	4.7%	5.8%	
Slightly advantaged	928	3.4%	4.7%	
Slightly disadvantaged	1,996	1.7%	3.5%	
Most disadvantaged	1,114	1.5%	3.6%	
**Proportion of patients Black (Non-Hispanic)**				0.35
0–25%	2,685	4.7%	10.1%	
25–50%	317	5.3%	8.6%	
50–75%	89	3.6%	8.5%	
75–100%	41	3.1%	8.9%	
**Proportion of patients Hispanic**				0.62
0–25%	3,088	4.7%	9.9%	
25–50%	43	6.0%	10.1%	
50–75%	<11^a^	0.0%	0.0%	
75–100%	0	–	–	
**Proportion of patients Asian**				0.25
0–25%	3,104	4.7%	9.9%	
25–50%	24	6.3%	13.7%	
50–75%	<11	15.0%	13.2%	
75–100%	<11	0.0%	0.0%	

a *Cells expressing imprecise measurements are due to CMS small cell suppression policy. (https://www.hhs.gov/guidance/document/cms-cell-suppression-policy). Center for Medicare and Medicaid Services has set minimum cell sizes to protect the confidentiality of Medicare and Medicaid beneficiaries by avoiding the release of information that can be used to identify individual beneficiaries and therefore, we report cell sizes that comply with the requirements. When a small cell is present, two cells in the category must be masked to eliminate the mathematical derivation of the small cell*.

In the multivariable, mixed-effects logistic regression analysis of the final study year cohort (2014), patient characteristics independently associated with reduced frequency of IVT use were advanced age (≥86 years), Black and North American Native race-ethnicity, fewer comorbid health conditions, arrival by private vehicle/walk-in (rather than EMS transport), and residence in the Southern region (vs. Western region) of the United States.

Hospital characteristics independently associated with reduced IVT use were as follows: Non-stroke center, located in the most socioeconomically disadvantaged areas (vs. most advantaged), and smaller size hospitals (Table 4).

**Table 4:** Multivariable analysis of patient and hospital characteristics associated with IVT in Year 2014 cohort (*N* = 28,434).

**Covariate (Reference group)**	**Adjusted** **odds** **ratios**	**95% Confidence** **interval**		***P*** **Value**
		**Lower**	**Upper**	
**EMS Utilization (Non-EMS transportation)**				
Arrival by EMS	2.67	2.38	3.00	<0.001
**Sex (Female)**				
Male	1.08	0.98	1.18	0.10
**Age (66 to 75)**				
76 to 85	0.92	0.82	1.02	0.11
86 and above	0.74	0.65	0.83	<0.001
**Race-ethnicity (white, Non-Hispanic)**				
Black, Non-Hispanic	0.73	0.61	0.87	<0.001
Asian	0.89	0.63	1.27	0.53
Hispanic	1.15	0.81	1.63	0.43
American native	0.10	0.01	0.73	0.02
Other	0.83	0.55	1.28	0.41
**Stroke center certification (Non-stroke center)**				
Treated at a certified center	1.96	1.68	2.29	<0.001
**Geography (Urban)**				
Rural	0.88	0.77	1.00	0.05
**Charlson comorbidity index (Score** ** < = ** **1)**				
Charlson score ≥ 2	1.35	1.12	1.64	<0.001
**Hospital ADI group (most disadvantaged)**				
Most advantaged	1.27	1.05	1.54	0.01
Slightly advantaged	1.07	0.91	1.27	0.41
Slightly disadvantaged	0.94	0.80	1.11	0.48
**Census region (south)**				
Midwest	1.05	0.91	1.21	0.54
Northeast	1.08	0.93	1.26	0.30
West	1.21	1.02	1.43	0.03
US territories	0.44	0.06	3.40	0.43
**Hospital size (0 to 100 beds)**				
101 to 200 beds	2.29	1.65	3.20	<0.001
201 to 300 beds	2.82	2.03	3.91	<0.001
301 to 400 beds	3.17	2.28	4.42	<0.001
401 to 500 beds	2.86	2.02	4.06	<0.001
More than 500 beds	3.36	2.42	4.66	<0.001
**Race-Ethnicity patient proportions at hospital (white, Non-Hispanic)**				
Black, Non-Hispanic	0.84	0.56	1.27	0.42
Asian	1.84	0.49	6.94	0.37
Hispanic	1.81	0.50	6.53	0.37

## Discussion

This study of a national sample of Medicare beneficiaries in the United States found a steady and substantial increase in the frequency of IVT treatment over the 8-year study time period, with the treatment rate nearly tripling, from 1 in every 36 acute ischemic stroke patients in 2007 to 1 in every 13 acute ischemic stroke patients in 2014. Treatment rates increased across all patient and hospital characteristic groups, but in the final year of the study period disparities were still evident, with lower rates of IVT use in patients of highly advanced age, Black and North American Native race-ethnicity, treatment at a rural hospital, and treatment at a hospital located in the most socioeconomically disadvantaged areas. Additional features associated with lower rates of IVT use were fewer comorbid health conditions, arrival by private vehicle/walk-in, treatment at a hospital in the South, a hospital of smaller size, and a hospital not certified as a stroke center (thrombolysis-capable).

This study focuses on data from the early to mid-part of the last decade. This is a time period that both has not been consistently studied in a large representative sample of stroke patients and provides valuable insight into treatment trends that can inform current decision-making. While there may have been changes subsequent to the data analyzed here, history has shown that shifts in treatment patterns tend to be slow and steady, if not altogether resistant to change without external influence. Furthermore, data from the current year and the year prior are often not complete in national databases, which limits the ability to study immediate trends.

The finding in the current study of increasing IVT treatment rates over time is consistent with, and importantly extends, prior investigations. Earlier studies of national administrative databases in the United States found that IVT treatment rates increased from <1% in 2001 to 2.4–2.6% in 2004–2006 and to 5.2% in 2009 (4, 5, 26). The present study finds that this secular improvement in therapy delivery subsequently continued, with findings confirming the increase from 2007 to 2009 and demonstrating further advance from 2010 to 2014. Reports of treatment frequencies from the Get with the Guidelines–Stroke nationwide clinical registry and the Centers for Disease Control and Prevention (CDC) multistate registry also showed increased treatment rates preceding and in the early portion of the current study time period, from 2003 to 2011 (6, 27). However, though the directional evolution was similar, reported treatment rates were higher in these registry reports than in analyses of administrative data. This difference likely reflects the combined effects of registries disproportionately including certified stroke centers that have higher treatment rates and of administrative datasets failing to identify some treatment patients due to under-coding (28). Although the data sources and case-identification methods are very disparate, the analysis presented here confirms and validates these previous results showing an increase in IVT over time in a more representative national sample of stroke patients.

The current study's findings regarding race-ethnic, age, and regional differences in the use of IVT is also largely consistent with prior studies and shows that these differences persisted in more recent years despite the broad increase in treatment across all patient groups. Studies of US national administrative databases found lower IVT treatment in patients who were Black, older, and residing in rural areas during 2004–2010, a period preceding and overlapping with the early portion of the current study time period (29–31). Conversely, the current study did not find reduced rates of IVT rates in Hispanic or Asian patients, two race-ethnic groups for whom prior investigations have provided inconsistent findings regarding presence or absence of reduced treatment frequencies. While our study is limited to patients >65 years of age, other studies have reported age, sex, and race disparities in thrombolysis treatment. A study of the Get With The Guidelines-Stroke registry reported that for patients ≤ 40 years of age with acute ischemic stroke hospitalized between January 2009 and September 2015, thrombolysis use was higher than those over 40 years of age (32). A retrospective analysis of Asian American and white patients admitted with a primary diagnosis of acute ischemic stroke to hospitals participating in the Get With The Guidelines-Stroke (GWTG-Stroke) program between 2004 and 2016 showed that Asian Americans were less likely to receive thrombolysis treatment (0.95, *p* = 0.003) even though the strokes were more severe (33). Findings from the (Florida-Puerto Rico Collaboration to Reduce Stroke Disparities) registry for the time period 2020–2015 showed that among patients with mild stroke, disparities existed in thrombolysis treatment for stroke. Younger patients, white race, lower risk of vascular disease, and less vascular risk factors, higher stroke severity were more likely to receive thrombolysis, while women were less likely (34, 35).

Our study also provides additional confirmatory and more contemporary evidence regarding the importance of pre-hospital processes and hospital resources and qualifications in the delivery of IV thrombolytic therapy. Patients arriving by EMS and at hospitals certified as facilities reliably capable of thrombolytic therapy delivery were more likely to receive IVT treatment. These same associations were found in earlier time periods in prior studies of regional and national registry data (6, 12, 36, 37). But, to our knowledge, this is the first study to confirm the importance of these factors in a more fully representative national administrative dataset. While the mechanism of this remains to be confirmed, we propose that EMS arrival ensures prompt attention at the receiving facility and pre-notification from the field can accelerate stroke team response and brain imaging availability upon arrival (38). Stroke center certification ensures that hospitals have sufficient resources to reliably deliver thrombolytic therapy and are engaged in continuous quality improvement to achieve best treatment rates.

Previous research has shown an association between higher Charlson's comorbidity index scores and poor outcomes in stroke patients. Among older adults hospitalized for acute stroke, higher comorbidity index (Charlson score ≥ 2) was associated with greater length of stay, treatment costs, and mortality (39). It has been shown that every 1-point increase in Charlson score was independently associated with poor outcomes and higher mortality, specifically a 15% increased odds of a poor outcome at discharge and a 29% increased odds of death by 1 year (40). Yet despite the confirmation of the association of Charlson score with outcomes and mortality, there has not been an examination of the association of this measure with the likelihood of thrombolysis treatment. In this study, we report increased use of thrombolytic treatment in patients with higher Charlson Score. This could be because a higher Charlson score may also be associated with stroke severity. However, our database does not include NIH Stroke Scale/Score (NIHSS) and therefore we were unable to confirm the contributions of NIHSS to this observed effect in the current study. This observation could be explored in the future using other databases that include NIHSS.

One additional novel finding of this study is the independent association between neighborhood socioeconomic status where the hospital is located and treatment patterns: patients brought to hospitals located in the least advantaged communities are less likely to receive IVT treatment. This finding likely reflects both fewer resources available to hospital facilities in socioeconomically disadvantaged facilities and also the effects of neighborhood income, quality of life, and employment opportunities upon knowledge of and response to stroke warning signs. This finding is consistent with a study of one Texas county that found an independent influence of neighborhood of residence upon use of EMS for stroke presentation (41).

### Limitations

This was an observational study and as such cannot determine causal relationships between patient or hospital characteristics and IVT use. Additional limitations include the following: first, the study does not include patients younger than 66 years, and therefore, results may not be generalizable to the younger population. Second, the study was performed using administrative databases in which coding may not identify all cases of thrombolytic treatment (27). However, the reliability of the findings is supported by similar observations in nationwide registries that use active case ascertainment and the current investigation extends the registry studies by including a more representative sample of all treating hospitals in the United States. Third, the current study could not control for presenting stroke severity or time since onset of presentation as these data were not recorded in administrative databases during the study period. Accordingly, the current study provides insight into patterns of IVT use among all acute ischemic stroke patients rather than among likely thrombolysis-eligible patients. The recent addition of the National Institutes of Health Stroke Scale as a date element in the US Medicare dataset will help to address this aspect in future studies (42).

Finally, our primary outcome is intravenous alteplase use (thrombolysis) use. In this manuscript, we did not additional process outcomes such as emergency department arrival to CT scan or arrival to thrombolysis due to absence of time stamps in administrative database. Also, we did not explore the effect of thrombolysis on patient outcome such as 90 day functional dependence or mortality since the focus of this manuscript is on treatment outcome and not patient-outcomes. Our future work will report patient outcomes and their relationship to prehospital and hospital predictors.

### Conclusions

Between 2007 and 2014, the frequency of IVT use for patients with acute ischemic stroke increased substantially, nearly tripling, though disparities persisted including less frequent treatment in patients of very advanced years, Black race-ethnicity, and rural residence. These findings can inform ongoing efforts to optimize the delivery of IVT to acute ischemic stroke patients nationwide.

## Data Availability Statement

Publicly available datasets were analyzed in this study. This data can be found at: https://www.cms.gov/Research-Statistics-Data-and-Systems/Files-for-Order/LimitedDataSets/MEDPARLDSHospitalNational.

## Ethics Statement

The studies involving human participants were reviewed and approved by Stanford University Institutional Review Board. Written informed consent for participation was not required for this study in accordance with the national legislation and the institutional requirements.

## Author Contributions

PG, AWT, and TM: had full access to all of the data in the study and take responsibility for the integrity of the data and the accuracy of the data analysis. TM, AWT, LM, and PG: acquisition, analysis, or interpretation of data. AA-RT, SR, TM, AWT, and PG: drafting of the manuscript. TM and AWT: statistical analysis. PG: obtained funding and supervision. SR: administrative, technical, or material support. All authors: concept and design and critical revision of the manuscript for important intellectual content. All authors contributed to the article and approved the submitted version.

## Funding

This project was funded under grant number R01HS026207 from the Agency for Healthcare Research and Quality (AHRQ), U.S. Department of Health and Human Services.

## Author Disclaimer

The authors are solely responsible for this document's contents, findings, and conclusions, which do not necessarily represent the views of AHRQ. Readers should not interpret any statement in this product as an official position of AHRQ or of the U.S. Department of Health and Human Services.

## Conflict of Interest

JS is an employee of the University of California. The University of California has patent rights in retrieval devices for stroke. The University of California Regents received payments on the basis of clinical trial contracts for the number of subjects enrolled in multicenter clinical trials sponsored by Medtronic, Stryker, Cerenovus, BrainsGate, and Boehringer Ingelheim (prevention only); JS served as an unpaid site investigator under these contracts. JS serves as an unpaid consultant to Genentech advising on the design and conduct of the PRISMS trial; neither the University of California nor JS received any payments for this voluntary service. JS paid for his own travel. JS has received contracted hourly payments and travel reimbursement for services as a scientific consultant advising on rigorous trial design and conduct to Medtronic, Stryker, Cerenovus, BrainsGate, Boehringer Ingelheim (prevention only), Diffusion Medical, and Abbott. JS has received contracted stock options for services as a scientific consultant advising on rigorous trial design and conduct to Rapid Medical. JS has not participated as a medicolegal expert in any litigation regarding acute stroke management. The remaining authors declare that the research was conducted in the absence of any commercial or financial relationships that could be construed as a potential conflict of interest.

## Publisher's Note

All claims expressed in this article are solely those of the authors and do not necessarily represent those of their affiliated organizations, or those of the publisher, the editors and the reviewers. Any product that may be evaluated in this article, or claim that may be made by its manufacturer, is not guaranteed or endorsed by the publisher.
