# Supplementary material for: Lessons Learned From the Historical Trends on Thrombolysis Use for Acute Ischemic Stroke Among Medicare Beneficiaries in the United States
Source: Front Neurol. 2022 Mar 4;13:827965. doi: 10.3389/fneur.2022.827965 (PMC8931506; doi:10.3389/fneur.2022.827965)
Supplement: Supplementary file 1 [file Table_1.docx]

Supplementary Material

Supplemental Table 1: Characteristics of Patients Admitted Between in 2007 and 2014 by Treatment Status

|  | | | | |  |
| --- | --- | --- | --- | --- | --- |
|  |  |  |  |  |  |
|  | **Patients, No. %** | | | | |
| **Characteristics** | **No IVT N=216,420** | | **IVT N= 11,657** | | **P Value** |
|  | **N** | **%** | **N** | **%** |  |
| **Gender** |  |  |  |  |  |
| Male | 86,454 | 39.95% | 4,884 | 41.90% | 0.0405 |
| Female | 129,966 | 60.05% | 6,773 | 58.10% |  |
| **Age Group** |  |  |  |  |  |
| 66 to 75 | 63,669 | 29.42% | 3,711 | 31.83% | 0.0168 |
| 76 to 85 | 87,711 | 40.53% | 4,919 | 42.20% |  |
| 86 and above | 65,040 | 30.05% | 3,027 | 25.97% |  |
| **Race** |  |  |  |  |  |
| White | 184,825 | 85.40% | 10,242 | 87.86% | <.001 |
| Black | 22,087 | 10.21% | 890 | 7.63% |  |
| Asian | 3,142 | 1.45% | 199 | 1.71% |  |
| Hispanic | 3,527 | 1.63% | 200 | 1.72% |  |
| American Native | 783 | 0.36% | 21 | 0.18% |  |
| Other | 2,056 | 0.95% | 105 | 0.90% |  |
| **Charlson Comorbidity** |  |  |  |  |  |
| Charlson Score <=1 | 144,885 | 8.51% | 6,171 | 8.51% | <.001 |
| Charlson Score >= 2 | 71,535 | 33.05% | 5,486 | 47.06% |  |
| **Geography** |  |  |  |  |  |
| Urban | 173,552 | 80.19% | 10,043 | 86.15% | <.001 |
| Rural | 42,868 | 19.81% | 1,614 | 13.85% |  |
| **Census Region** |  |  |  |  |  |
| Midwest | 55,175 | 25.49% | 2,825 | 24.23% | <.001 |
| Northeast | 39,953 | 18.46% | 2,510 | 21.53% |  |
| West | 33,000 | 15.25% | 2,099 | 18.01% |  |
| South | 87,778 | 40.56% | > 4,212^1^ | > 36.13% |  |
| US territories | 514 | 0.24% | < 11 | < 0.51% |  |
| **EMS Utilization** |  |  |  |  |  |
| Ambulance Users | 84,181 | 38.90% | 2,133 | 18.30% | <.001 |
| Other Means of Transportation | 132,239 | 61.10% | 9,524 | 81.70% |  |
|  |  |  |  |  |  |

^1^Cells expressing imprecise measurements are due to CMS small cell suppression policy. (<https://www.hhs.gov/guidance/document/cms-cell-suppression-policy>). Center for Medicare and Medicaid Services has set minimum cell sizes to protect the confidentiality of Medicare and Medicaid beneficiaries by avoiding the release of information that can be used to identify individual beneficiaries and therefore, we report cell sizes that comply with the requirements. When a small cell is present, two cells in the category must be masked to eliminate the mathematical derivation of the small cell.
